# Adolescent Compliance with anti-COVID Measures. Is It Related to Substance Use?

**DOI:** 10.1007/s11469-021-00751-4

**Published:** 2022-01-13

**Authors:** Joaquín Rodríguez-Ruiz, Izabela Zych, Vicente J. Llorent

**Affiliations:** 1 Department of Psychology, University of Cordoba, Avda. San Alberto Magno s/n. 14004, Cordoba, Spain; 2 Department of Education, University of Cordoba, Cordoba, Spain

**Keywords:** COVID-19, Compliance with measures, Licit substances use, Illicit substance use, Adolescents

## Abstract

Prevalence rates of compliance with anti-COVID measures have been widely studied, but little is known about this issue in early adolescence. Moreover, the relation between substance use and compliance with anti-COVID regulations is still unexplored. Thus, this study aimed to determine the level of compliance with anti-COVID measures by adolescents and the link between substance use and compliance with anti-COVID regulations. This was a cross-sectional study including 909 participants (*M*_age_ = 12.57; *SD* = 0.81). The most complied measure was mask-wearing, followed by avoiding hug/kiss friends and, finally, social distancing. All substance use negatively correlated with compliance with measures. However, strong alcohol and tobacco were the only substances significantly related to less compliance of anti-COVID measures after controlling for covariates. These results provide evidence about the relation between substance use and compliance with anti-COVID measures. Strategies addressed to decrease substance use could be effective to reduce behaviours associated with coronavirus transmission.

During the global pandemic caused by COVID-19, administrations of most countries adopted restrictive measures that, in many cases, have meant a radical change in people’s daily lives (Sohrabi et al., 2020). Lockdowns, social distancing and the uncertainty generated by the pandemic have triggered an increase in adverse psychological reactions in the population, such as stress, anxiety or depressive symptoms (Salari et al., 2020; Savolainen et al., 2021).

Some people use substances as a coping strategy against adverse psychological reactions (Sinha, 2008). This can be even more true in adolescence which is a period of deep biological changes that can trigger some emotional instability (Bailen et al., 2019). At the same time, Pardo et al. (2002) showed that drug use increases disinhibition and anti-normative behaviours. Thus, substance use can trigger undesirable behaviours that are incompatible with COVID-19 measures. Substance use and other antisocial behaviours, including rule-breaking, tend to form patterns (Nasaescu et al., 2020), and these problem behaviours could also be two manifestations of the same underlying construct. This can be especially true in adolescence, a period characterized by high risk-taking and sensation-seeking (Steinberg, 2004) that are further increased by substance use (Lane et al., 2004).

The COVID-19 pandemic is thus likely to contribute to an increase in the prevalence rates of substance use (Rehm et al., 2020), and substance use could worsen the pandemic because it could decrease citizens’ willingness to comply with COVID-19 rules. Limiting alcohol consumption has been included in some specific national and regional strategies against COVID-19, assuming that “alcohol is a psychoactive substance which inhibits judgement” and “the direct effects of alcohol impair consumers’ ability to comply with transmission control measures in hospitality settings” (Scottish Government, 2020). Nevertheless, the relation between substance use and compliance with COVID-19 rules still needs to be empirically studied. Policies have been developed to reduce substance use in order to prevent the spread of coronavirus. Among them, it has been banned to smoke in Spain when safe distance could not be kept (BOJA 51, 2020), and restrictions to the sale of alcohol at certain hours have been decreed (BOJA 16, 2021). Most of these policies have been based on intuitions and knowledge from related fields, but it still needs to be discovered if substance use is related to the adherence to anti-COVID measures. Thus, the current study focused on the relation between substance use and the compliance with the COVID-19 measured in a broad sample of adolescents.

Several studies focused on the compliance with the COVID-19 regulations. Among them, Wismans et al. (2020) analysed compliance with hygiene measures and social distancing. Overall, they found higher compliance with social distancing measures than with hygiene measures. They also found that males and younger participants scored lower both at hygiene and social distancing measures, but they generally found high percentages of adherence to anti-COVID measures. Another study with 18 to 87-year-old Chinese participants found that 96.4% reported wearing masks, 79.1% hand washing and 42.3% kept social distancing (Tong et al., 2020). A study with 14 to 22 years old young Canadians conducted by Yang et al. (2020) found that 60% highly complied with all measures. Females were more prone than males to adhere to health measures.

In a study conducted by Nivette et al. (2021), social distancing measures were more commonly followed than hygiene measures and males tended to comply less with COVID-19 measures. However, Oosterhoff and Palmer (2020) surveyed 13 to 18-year-old US adolescents and found that 87.8% disinfected hands daily, whereas 21.1% kept social distancing. Riiser et al. (2020) also found significant gender differences in 16–19 years Norwegian students: 80.9% boys versus 88.9% girls declared hand washing after socializing. In another study, overall scores of Jordan adolescents on attitudes towards anti-COVID measures were high, and compliance with measures was more prevalent among females and younger participants (Dardas et al., 2020).

The number of studies focused on compliance with anti-COVID measures in Spain is low. Among them, lower scores on compliance with anti-COVID measures, hand washing and social distancing were reported by women and young adults (18–25 years) compared to men and older adults (51–72 years). Young adults also tended to avoid wearing a mask (De la Vega et al., 2020). Barceló and Sheen (2020) also showed less mask-wearing in young adults. No gender differences were found regarding wearing masks by De la Vega et al. (2020), and Barceló and Sheen (2020). Thus, the number of studies focused on COVID-19 measures in adolescents in Spain and worldwide is low.

Substance use in adolescence has been widely studied with similar results among different reports where the most used substance is alcohol, followed by tobacco, cannabis and other illegal drugs —MDMA, inhalants, cocaine, etc.— (ESPAD Group, 2020; Johnston et al., 2021; Moreno et al., 2020; Observatorio Español de las Drogas y las Adicciones, 2020). Substance use is widely spread among children and adolescents, with a clear increase in prevalence rates across adolescence (Zych et al., 2020). Given high prevalence rates of substance use and its possible impact on adolescents’ behaviour, the relation between substance use and compliance with anti-COVID measures by adolescents should be further studied.

Scientific literature to date discovered an increase in patterns of substance use during the pandemic (Branquinho et al., 2020; Dumas et al., 2020; Gritsenko et al., 2020; Sun et al., 2020; Vidot et al., 2020). Some studies focused on the relation between substance use and the impact of the pandemic on mental health and wellbeing. Positive correlations have been found between the psychological impact of the pandemic and alcohol problems (Panno et al., 2020). Another study found that alcohol, tobacco and cannabis users reported higher levels of fear and concern related to COVID-19 in comparison to non-users (Rogers et al., 2020). Moreover, a report published by Petterson et al. (2020) estimated that, due to the pandemic, up to 154,000 deaths of despair could occur, including people who lose their lives as a consequence of alcohol and drug use. Hence, more research is needed to gain knowledge on the emotional impact of the pandemic among substance users.

Substance users are a risk population regarding COVID-19 infections. Given that coronavirus can be transmitted through droplets (Leung, 2021), tobacco use could increase the transmission. Other studies focused on the vulnerability to COVID-19 of alcohol (Wang et al., 2021), tobacco (Haddad et al., 2021) and other substances users (Wei & Shah, 2020). A literature review conducted by Dubey et al. (2020) suggested that substance abuse–related behaviours, such as sharing bottles and needles or spitting habits among tobacco users, can be a risk factor for coronavirus spreading. However, to the best of our knowledge, there are no studies to date focused on the level of compliance with anti-COVID measures by substance users.

## The Current Study

Adherence to anti-COVID measures has been studied since the beginning of the pandemic, but it is still necessary to discover factors related to compliance with COVID-19 regulations in adolescents. Moreover, a growing body of research relates substance use and vulnerability to COVID-19. However, there is a gap in knowledge regarding compliance with anti-COVID measures by people who use drugs. Substance use could be a risk factor because it provokes disinhibition and, in some cases, users share substances or materials (cigarettes, bottles, etc.). Nevertheless, the relation between adolescent substance use and compliance with anti-COVID measures still needs to be discovered. For these reasons, the objectives of the current study are as follows: (i) to explore the level of compliance with COVID-19 regulations by adolescents and (ii) to explore a possible link between adolescent substance use and adherence to anti-COVID measures.

## Methods

### Participants

The sample included 909 early adolescents (48.4% girls; 51.6% boys) from the province of Cordoba (Spain). Students were enrolled in 16 secondary education schools. Mean age of the participants was 12.57 years (*SD* = 0.81) and they were enrolled in Grade 1 and Grade 2 of Compulsory Secondary Education.

Participants’ responses were kept if they filled in at least 66% of the items of each questionnaire. The rate of participants who filled in more than 66% of the items was as follows: 96.37% substance use scale (*n* = 876), 96.59% intoxication scale (*n* = 878), 99.56% compliance of COVID-19 regulations scale (*n* = 905) and 98.13% emotional impact of pandemic (*n* = 892). Listwise, delation was used for the participants who did not fill in 66% of the items in each scale.

### Instruments

Substance use was measured with a *substance use* subscale (*Ω* = 0.94; *α* = 0.93), of the *Self-Reported Antisocial Behaviour Questionnaire* (SRA) designed and evaluated by Loeber et al. (1989). This subscale has seven items focused on having used beer, wine, strong alcohol, tobacco, cannabis, cocaine and other illicit drugs during the past school year, answered on a four-point Likert scale (1 = *never*; 2 = *yes, once*; 3 = *yes, twice*; 4 = *yes, more times*). A confirmatory factor analysis showed an adequate fit of the current data to the original factor structure of the questionnaire (*χ*^2^ = 3035.96, df = 485, *p* < 0.05, CFI = 0.93, NFI = 0.92, RMR = 0.06, RMSEA = 0.082, 90% *CI* = 0.079–0.085).

*Substance intoxication* was measured through three items created ad hoc for this study: Have you ever got drunk with alcohol? Have you ever got strongly stoned with any drug (excluding alcohol)? Have you ever drunk a lot and quickly to get drunk? The questionnaire was answered on a four-point Likert scale (1 = *never*; 2 = *yes, once*; 3 = *yes, twice*; 4 = *yes, more times*). This scale had an excellent reliability (*α* = 0.94, *Ω* = 0.94).

*Compliance of COVID-19 regulations* (*α* = 0.62, *Ω* = 0.64) was measured with three items created ad hoc: How often do you hug and/or kiss your friends since the beginning of the pandemic? How often do you wear a mask whilst you spend time with your friends since the beginning of the pandemic? How often do you keep social distancing whilst you spend time with your friends since the beginning of the pandemic? Participants responded according to the following Likert scale: 1 = *never*, 2 = *monthly*, 3 = *weekly*, 4 = *daily*, 5 = *all the time*.

*Emotional impact of the COVID-19 pandemic* was measured with one item (How frequently have you felt bad in relation to the COVID-19 pandemic?). The item was responded on a five-point Likert scale (1 = *never*, 2 = *monthly*, 3 = *weekly*, 4 = *daily*, 5 = *all the time*).

### Design and Procedure

This was a cross-sectional study that included a sample selected by convenience. Schools’ board directors were contacted to ask them for participation in the study. Participants were under 18, so parental consents were obtained. Data were collected in October and November 2020.

Before filling in the questionnaires, students were informed that their participation was voluntary, anonymous and that they could withdraw from the study at any time. In 55.6% of the schools, questionnaires were administered in a paper-and-pencil form, supervised by the research team. Some schools had COVID-19 restrictions and did not allow entrance of any person who was not a part of their school community. Thus, 44.4% (*N* = 404 participants) of the questionnaires were administered online. During the online administration, participants were supervised by their teachers, who received instructions from the research team. They were asked not to intervene in the administration process, not answer questions, and supervise the students to make sure that they filled in the questionnaire individually, anonymously, and in a silent environment. The study followed all the national and international ethics standards, and it was approved by the Ethics Committee of the University of Cordoba.

### Data Analyses

Descriptive analyses were performed to obtain percentages of compliance with anti-COVID measures and feeling bad about the pandemic. Spearman correlations were performed to explore relations among four variables including compliance with COVID-19 regulations, feeling bad about the pandemic, each substance use and intoxication. Mean differences regarding compliance with COVID-19 measures by sex and grade were tested using Student’s *t*-test analysis.

To discover which variables were uniquely related to compliance with COVID-19 regulations, after checking the assumptions of linearity, normality, homoscedasticity and independence of residuals, a lineal regression analysis was performed. Compliance with COVID-19 regulations was entered as the dependent variable, and sex, age, each substance use, intoxication and feeling bad about the pandemic were entered as independent variables. All these analyses were run using SPSS version 25 software.

## Results

### Participants’ Characteristics

Among the participants, 447 (49.2%) were enrolled in Grade 1 of Compulsory Secondary Education, and 462 (50.8%) were enrolled in Grade 2. The ethnicity of the sample was 95% Majority Spanish, 1.6% Roma, 0.1% Sub-Saharan, 0.6% Latin American, 0.5% Maghrebi and 2.3% other. Participants were also asked about socio-economic status of their families, and 94.2% of them self-identified as being neither rich nor poor, 0.9% classified their families as poor, 4.3% as rich and 0.6% as very rich.

### Descriptive Analyses

**Fig. 1:**
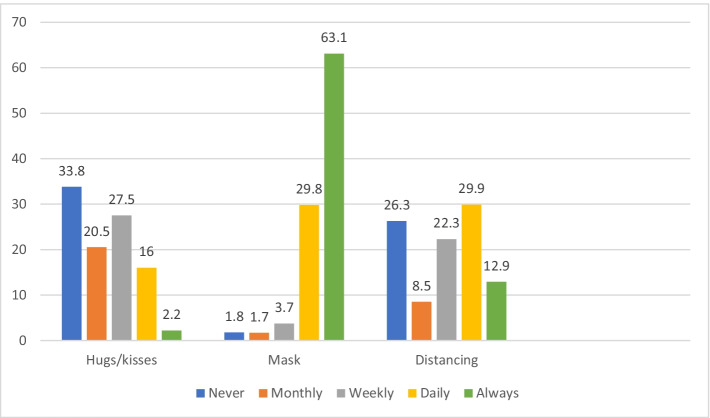
Percentages of compliance with COVID-19 regulations

Frequencies of compliance with each anti-COVID measure are described in Fig. 1. The highest compliance was reported regarding *mask-wearing* (63.1%). *Social distancing* was always kept by 12.9%, and almost a third of the sample reported that they were incompliant with this regulation daily. Moreover, 66.2% *hugged and/or kissed* their friends at least monthly.

### Relation Between Compliance with COVID-19 Regulations and Other Variables

Table 1 shows correlations among *compliance with COVID-19 regulations*, *feeling bad about the pandemic*, each *substance use* and *intoxication*. *Feeling bad about the pandemic* was not significantly related to *compliance with COVID-19 regulations*. However, the use of any studied *Substance* and *Intoxication* was related to less *compliance with COVID-19 measures*.

**Table 1:** Relations among the compliance with COVID-19 regulations, substance use, intoxication and feeling bad about the pandemic

	1	2	3	4	5	6	7	8	9
1. Compliance of COVID-19 regulations									
2. Feeling bad about the pandemic	.038								
3. Beer use	− .225^**^	.039							
4. Wine use	− .160^**^	.053	.620^**^						
5. Strong alcohol use	− .303^**^	.021	.544^**^	.410^**^					
6. Tobacco use	− .226^**^	.016	.422^**^	.346^**^	.527^**^				
7. Cannabis use	− .115^**^	.061	.223^**^	.231^**^	.291^**^	.359^**^			
8. Cocaine use	− .070^*^	.063	.082^*^	.141^**^	.186^**^	.308^**^	.499^**^		
9. Other strong drug use	− .088^*^	.042	.114*^*^	.162^**^	.227^**^	.284^**^	.712^**^	.781^**^	
10. Intoxication									
	− .207^**^	.036	.393^**^	.295^**^	.553^**^	.508^**^	.400^**^	.307^**^	.316^**^

^*^p < .05, **p < .01

### Grade and Sex Differences Regarding Compliance with COVID-19 Regulations

*Compliance with COVID-19* regulations was significantly higher in Grade 1 (*M* = 2.83; *SD* = 0.75) than in Grade 2 (*M* = 2.58; *SD* = 0.87) according to the *t*-test (*t* = 4.56; *d* = 0.31; *p* < 0.01). Boys (*M* = 2.78; *SD* = 0.79) showed higher level of compliance when compared to girls (*M* = 2.62; *SD* = 0.85). These mean differences were also significant (*t* =  − 2.81; *d* = 0.19; *p* = 0.01).

### Unique Relations with Compliance with COVID-19 Regulations

Linear regression analysis showed that being female and older, as well as strong alcohol and tobacco use, was significantly related to lower *compliance with COVID-19 regulations*. Details can be seen in Table 2. Moreover, interactions between sex and feeling bad about the pandemic (*B* = 0.02; *SE* = 0.04), sex and strong alcohol use (*B* =  − 0.02; *SE* = 0.08), as well as sex and tobacco use (*B* =  − 0.03; *SE* = 0.10) in relation to compliance with COVID-19 measures were tested, but they were not significant (*p* > 0.05).

**Table 2:** A regression analysis including substance use, sex, age, feeling bad about the pandemic as predictors of compliance with COVID-19 regulations

	Beta	Standard error	*t*	*P*
Sex	.086	.055	2.534	.011
Age	− .129	.036	− 3.695	< .001
Feeling bad about the pandemic	.053	.021	1.571	.117
Beer use	− .014	.062	− .297	.767
Wine use	− .047	.062	− 1.100	.271
Strong alcohol use	− .225	.065	− 4.323	< .001
Tobacco use	− .099	.068	− 2.168	.030
Cannabis use	− .032	.186	-.549	.583
Cocaine use	.034	.206	.578	.563
Other strong drug use	− .027	.305	− .435	.663
Intoxication	.075	.144	1.532	.126

### Discussion

Since the beginning of the pandemic, different measures have been decreed by administrations in order to avoid the spread of coronavirus. In Spain, following the guidelines by the WHO, people must keep social distancing and wear face masks in public spaces or when social distancing is not possible to keep (Han et al., 2020).

As a public health concern, the level of compliance with COVID-19 regulations has been studied in several countries (Nivette et al., 2021; Tong et al., 2020; Wismans et al., 2020; Yang et al., 2020). However, there are still many gaps in knowledge regarding compliance with COVID-19 regulations. The majority of studies has been conducted with adults (Barceló & Sheen, 2020; De la Vega et al., 2020), and research with adolescents is still necessary. Moreover, changes in substance use behaviours have been reported during the pandemic and these changes have been related to a negative experience of the pandemic (Gritsenko et al., 2020; Sun et al., 2020; Vidot et al., 2020). However, there is a gap in knowledge regarding the relation between substance use and compliance with COVID-19 regulations. Thus, this study aimed to discover the level of compliance with COVID-19 regulations and its relation to substance use in adolescents.

In relation to the first objective, the highest compliance was found regarding mask-wearing. Adolescents seem to be aware of the importance of face masks, given that less than 2% reported never wearing it. This is congruent with the results of Tong et al. (2020), who also found that mask-wearing was the most respected rule, although their percentage of compliance was higher. A possible explanation to such difference could be that the culture of mask-wearing is more extended among individuals who live in Asian countries for different reasons, such as pollution or other diseases (Choi & Lee, 2021).

In our study, social distancing was reported to be kept always only by 12.9%. Other studies with adult samples found higher percentage of compliance with social distancing (Nivette et al., 2021; Tong et al., 2020; Wismans et al., 2020), but Oosterhoff and Palmer (2020) pointed out that just around a fifth of adolescents complied with this recommendation. Moreover, only one out of three students in our study reported never kissing and/or hugging their friends. Sports activities and other games or a low number of spacious places to socialise could explain these overall low levels of compliance with COVID-19 regulations. Thus, educators should promote keeping social distancing and limiting physical contact among students in order to prevent the transmission of coronavirus.

Sex and grade differences regarding compliance with COVID-19 regulations were reported by the participants of our study. Previous studies found that young people were more likely to skip COVID-19 norms (Barceló & Sheen, 2020; De la Vega et al., 2020; Wismans et al., 2020). However, we found that compliance with COVID-19 regulations was higher in younger adolescents. This is congruent with Dardas et al. (2020) who found similar results in Jordan adolescents. Given these results, the relation between age and adherence to COVID-19 measures may not be linear. It is possible that younger adolescents preserve some moral values from childhood and their compliance with rules is higher in comparison to older adolescents. Middle and late adolescents tend to challenge rules and involve in risky behaviours (Steinberg, 2004). These trends usually disappear as young people mature and compliance with measures increases in adulthood (Gibbs, 2020). It is also possible that parental supervision of younger adolescents is higher than the supervision of older adolescents. It is, therefore, possible that adolescents who are supervised more closely are more compliant with COVID-19 regulations.

In our study, boys reported higher compliance with COVID-19 measures than girls. The opposite results have been reported by adult samples in other contexts (Nivette et al., 2021; Yang et al., 2020), as well as by older adolescents (Riiser et al., 2020) and adolescents aged 12–18 (Dardas et al., 2020). A plausible explanation for this sex divergence with previous research could be the developmental stage of participants. Our study focuses on early adolescence. It is known that girls usually mature earlier than boys (Lim et al., 2015) and, thus, girls in our sample could display behaviours in which boys involve later. Moreover, a kiss on each cheek is a casual and common greeting among females in Spain, but not among males. Thus, it is possible that this sex difference reflects this part of the Spanish culture.

Although changes in patterns of substance use during the pandemic have been reported in previous studies (Branquinho et al., 2020; Dumas et al., 2020), together with a negative perception of the pandemic among substance users (Dubey et al., 2020; Panno et al., 2020; Rogers et al., 2020), there are gaps in knowledge regarding the compliance with COVID-19 regulations among substance users. Precisely, the second objective of this study addressed this gap in knowledge. Although the use of any of the studied substances negatively correlated with adherence to COVID-19 regulations, strong alcohol and tobacco were the only substances that were uniquely related to lower compliance with COVID-19 measures after controlling for covariates. The explanation could be threefold. First, disinhibition provoked by alcohol could cause skipping these rules by the adolescents. Second, it has been found that adolescents tend to engage in more than one antisocial behaviours at the same time (Nasaescu et al., 2020). In this case, substance use and skipping rules can be considered two antisocial behaviours. Third, drug use itself can involve taking off masks and less social distancing at tables or smaller places. Nevertheless, the use of illegal substances was not related to compliance with COVID-19 regulations in the regression analysis. A possible reason is that substances such as cannabis or cocaine are not yet widely accepted by the peer group in early adolescents. Thus, students at this age who use this kind of substances may do it with a limited number of people instead of using them as social behaviour.

The present study has several strengths. Among them, it focuses on an understudied topic which explored the link between substance use and compliance with COVID-19 regulations. Moreover, it reported the level of compliance with COVID-19 regulations by Spanish adolescents. To the best of our knowledge, this is the first study in which these variables are studied and related with this population. Most of the previous studies on COVID-19 were conducted online or by phone, whereas participants in this study were supervised during data collection. Undoubtedly, this improves the quality of the data. However, there are also some limitations. The design is cross-sectional, so the order of appearance of phenomena cannot be established. It is possible that substance use causes rule-breaking related to COVID-19 regulations. It is also possible that substance use and non-compliance with COVID-19 measures are associated with one another solely because of their relation to a third variable (e.g. externalizing behaviours). Furthermore, the sample was selected by convenience, and future studies with representative samples should confirm our results. As data were collected using self-reports, a possible presence of social desirability bias should be taken into account. Future projects should study these variables using longitudinal designs and representative samples. Moreover, compliance with COVID-19 regulations should be related to other antisocial behaviours, such as bullying, cyberbullying or cyberhate.

Despite these limitations, this study has important implications for policy and practice. These results provide a framework to policymakers when regulations about substance use to reduce the transmission of the virus are made. It has been confirmed that tobacco and alcohol use are related to a lower level of compliance with COVID-19 regulations. Thus, the usefulness of policies limiting substance use during the pandemic seems to be confirmed, particularly during the outbreaks of the pandemic. Moreover, families should supervise their adolescent children, so they are not involved in substance use, especially during the pandemic. Although it is not allowed to sell alcohol and tobacco to minors in Spain, substance use in adolescents is common (Rodríguez-Ruiz et al., 2021). Many adolescents reported noncompliance with COVID-19 measures. Thus, complying with anti-COVID regulations and reducing substance use should be further promoted by schools and taken into account by policymakers.
